# Policy landscape analysis for fruits and vegetables in four low- and middle-income countries through a food systems approach

**DOI:** 10.1371/journal.pone.0331287

**Published:** 2025-09-19

**Authors:** Elaine Q. Borazon, Samali Perera, Nestor Alokpaï, Mario Sibamenya Venance, Erica Reeve, Jody Harris, Takwa Tissaoui, Anne Marie Thow

**Affiliations:** 1 International Graduate Program of Education and Human Development, College of Social Sciences, National Sun Yat-sen University, Kaohsiung City, Taiwan; 2 Leeder Centre for Health Policy, Economics and Data, School of Public Health, The University of Sydney, Sydney, Australia; 3 School of Rural Sociology and Agricultural Extension, University of Agriculture, Porto-Novo and Ketou, Benin Republic; 4 Buhigwe District Council, Division of Health, Social Welfare and Nutrition Services, Buhigwe, Kigoma-Tanzania; 5 Institute for Health Transformation, Global Centre for Preventive Health and Nutrition, School of Health and Social Development, Faculty of Health, Deakin University, Geelong, Australia; 6 World Vegetable Center, Nakhon Pathom, Thailand; Lusofona University of Humanities and Technologies: Universidade Lusofona de Humanidades e Tecnologias, PORTUGAL

## Abstract

Low fruit and vegetable intake is a significant nutritional issue in low- and middle-income countries, where resolving all forms of malnutrition remains a pressing challenge. Nutritional status is influenced by many dietary factors, and enhancing fruit and vegetable consumption offers significant health benefits and contributes to overall dietary quality. The study aimed to examine the policy landscape for fruits and vegetables across the food system in four low-middle income countries (Benin, Sri Lanka, Tanzania, the Philippines) and identify opportunities to strengthen food systems policies to enhance fruit and vegetable consumption as part of a comprehensive strategy to improve nutrition outcomes. A comparative qualitative analysis of policy documents (n = 14 [Benin], 18 [Sri Lanka], 30 [Tanzania], 55 [the Philippines] relevant to fruits and vegetables at the national and subnational levels, framed by a food systems framework, was conducted. A modified SWOT analysis was then conducted to formulate strategic policy recommendations to improve consideration of fruits and vegetables in policies tackling food system aspects. The analysis revealed specific opportunities for strengthening policy prioritization of fruits and vegetables across the food system such as multisectoral collaboration, policy integration between the national and subnational level, sustainability and resilience, and inclusion and equity. By addressing factors influencing fruit and vegetable intake within a dynamic food system, these countries and others facing challenges to the consumption of fruits and vegetables can effectively promote diet quality, improve food security, and address all forms of malnutrition through better policy design and particularly implementation.

## Introduction

Inadequate fruit and vegetable intake is associated with a loss of an estimated 16 million disability-adjusted life years globally from 2000-2019, which accounts for approximately 1% of the total, and 1.7 million deaths (equivalent to 2.8% of all deaths worldwide) [1]. Fruit and vegetable intake globally remains below the recommended intake of at least 400 grams/day by the World Health Organization [2–5]. It is lowest on average in low- and middle-income countries (LMICs), with significant gradients across economic development levels: mean daily consumption of 2.14 servings in low-income countries, 3.17 servings in lower-middle-income countries, 4.31 servings in upper-middle-income countries, compared to 5.42 servings in high-income countries [6,7]. These lower intake levels in LMICs are accompanied by a high prevalence of malnutrition and micronutrient deficiencies [2,8,9]. Conversely, adequate consumption of fruits and vegetables is associated with reduced risk of cardiovascular diseases [10], hypertension [11], oxidative stress [12], and cognitive malfunction [13], contributing to overall dietary quality and health [14–16].

Consumption patterns of fruits and vegetables worldwide are influenced by a complex interplay of factors beyond policy in food systems [5,16,17]. On the consumer side, there is often a lack of affordability of fruits and vegetables [6], in contrast to the affordability and availability of less healthy foods and beverages [18]. On the supply side, agricultural systems have undergone process intensification towards better efficiency leading to specialization on crops providing better yields – but at the expense of diversification of production [19], thus increasing the availability but in many cases reducing the diversity of fruits and vegetables in the marketplace. Moreover, agricultural productivity does not necessarily translate to demand fulfilment at the retail level or improvement in vegetable availability to the consumers, which are also influenced by post-harvest and supply chain capacities [20]. Policy affecting both supply and demand sides of food systems, and the food environments where these meet, is a significant factor in shaping healthy diets.

These interconnected challenges in fruit and vegetable access and availability highlight the need to understand and address the broader food system context in which they occur. To conceptualize these relationships more comprehensively, a food system is defined as “all the elements (environment, people, inputs, processes, infrastructures, institutions, etc.) and activities that relate to the production, processing, distribution, preparation and consumption of food, and the outcomes of these activities—namely nutrition and health status, socio-economic growth, and equity and environmental sustainability” [21]. While these elements play an important role in ensuring the delivery of high-quality diets, they are currently failing to provide healthy diets to all people in LMICs [22]. The determinants of intake at the population level are multifaceted and interconnected within a dynamic food system, which shapes the overall consumption patterns of fruits and vegetables, and addressing these determinants entails a comprehensive approach at multiple levels [4,23,24].

Comprehensive and coherent policies related to food and agriculture are critical to transform food systems and improve availability of healthier foods, including fruits and vegetables [25–27]. The influence of policies and regulations on supply, prices, safety, nutritional composition, and information received by consumers (among other factors) ultimately affect consumer choices and nutritional quality of diets [28–30]. The NOURISHING framework, for example, serves as comprehensive policy approach to address key domains of action [31]. We hypothesize that well-coordinated, context-specific policies addressing multiple domains of the food system simultaneously will be more effective in increasing fruit and vegetable consumption than isolated interventions targeting single aspects of supply or demand. Further, we posit that current policy landscapes in many LMICs exhibit significant gaps and/or lack of coherence across sectors that limit their effectiveness in promoting fruit and vegetable consumption.

However, the identification of specific policy measures across the range of sectors relevant to food systems transformation has proved challenging; each of these sectors has its own context and existing policy landscape, and points of leverage need to be considered within the policy landscape as a whole, across contexts, and across the food system [32,33]. Policy interventions can improve fruit and vegetable outcomes across various food system domains. For instance, fruit and vegetable incentive programs have shown a positive effect on both fruit and vegetable consumption and purchase patterns [34]. In schools, direct provision policies that specifically targeted fruits and vegetables have effectively increased fruit and vegetable consumption among children [35]. Moreover, agricultural policies supporting extension services for fruit and vegetable production have demonstrated success in reducing technical efficiency gaps leading to higher productivity than cereal crops [36]. Furthermore, targeted strategies to increase the density of food stores offering fruits and vegetables can lead to measurable improvements in dietary outcomes [37].

Beyond these sector-specific interventions, policies that explicitly integrate nutrition objectives—such as promoting fruit and vegetable intake—have demonstrated measurable effects on population health. For example, consumption of fruits and vegetables has been consistently associated with improved mental health outcomes across a majority of studies [38] and has been associated with a lower risk of adiposity [39]. Moreover, a meta-analysis has shown that adequate consumption of fruits and vegetables, in combination with other healthy dietary components, is linked to a reduced risk of all-cause mortality [40]. These findings show the role of fruit and vegetable intake as a modifiable risk factor for addressing the global burden of non-communicable diseases.

While policies have the potential to improve fruit and vegetable consumption, it remains unclear to what extent national food system policies in LMICs prioritize fruits and vegetables as strategic objectives. We hypothesize that limited prioritization stems from gaps in policy coherence, weak integration across sectors, and insufficient alignment with nutrition goals.

This study is motivated by the need to move from broad global recommendations to actionable, context-specific policy measures. Strengthening the nutrition-health nexus in food policy requires intentional alignment of interventions with clearly defined dietary and health goals, supported by monitoring systems that track nutritional outcomes.

This study aims to examine the policy landscape related to fruits and vegetables across the food system in four LMICs and to identify opportunities for strengthening multisectoral policy action. It is guided by the central research question: How can food system policies be better designed and integrated to effectively increase fruit and vegetable consumption in low- and middle-income countries? Given the evidence that policies across the food system are needed for improving outcomes, conducting a comparative analysis of different policy approaches will give much needed insight into how these broad recommendations for food system-wide policies are, and can be operationalized in practice, providing learnings for both these four countries and other jurisdictions seeking to implement food system-wide policies to support fruit and vegetables.

## Methods

### Theoretical frameworks

The research was informed by the High Level Panel of Experts (HLPE) food systems framework [21], which provides a comprehensive structure for analyzing how different food system components, including policy, can influence dietary outcomes. The HLPE framework conceptualizes the food system as interconnected domains spanning from production to consumption, including technology and policy drivers, food supply chains, food environments, and consumer behavior. We operationalized the HLPE food systems framework for this study in two phases. First, we identified five key domains within the food system where policies significantly impact fruit and vegetable availability, accessibility, and consumption: (1) biodiversity, genetic innovation and seed systems; (2) sustainable production systems; (3) postharvest and markets; (4) food environments; and (5) consumer behaviour. For each domain, we mapped the relevant policy sectors and documents in each of the four countries. Second, we selected specific policy areas within each part of the food system which are known to be key to supportive action on fruits and vegetables. For example, under biodiversity, we analyzed policies related to: (1) conservation and use of genetic resources; (2) crop improvement and plant breeding; (3) seed systems, seed production, seed policy and seed trade; and (4) consideration of fruits and vegetables as a part of biodiversity. The four LMICs selected for this study – Benin, Sri Lanka, Tanzania, and the Philippines – represent diverse contexts within which to examine how food system policies for fruits and vegetables are implemented in practice (see detailed descriptions below). These countries were selected based on their varying stages of food system transformation, different policy approaches to fruits and vegetables, and contrasting nutritional challenges. Applying the HLPE framework in this context allowed for a structured cross-country comparison of how fruit and vegetable policies are conceptualized, positioned, and implemented within broader food systems, thus allowing us to identify gaps and opportunities for integrated policy recommendations. Our analytical approach was guided by a team of experts involved in a broader program of work researching fruits and vegetables for healthy diets. This work is situated within the Fruit and Vegetables for Sustainable Healthy Diets (FRESH) initiative, which aims to increase fruit and vegetable intake to improve diet quality and nutrition, while also enhancing livelihoods, empowering women and youth, and mitigating environmental impacts [41].

We also drew on the theories of policy learning and transfer to inform the development of the extraction matrix, based on what has been found to be useful to inform learning in other contexts (both across time and space). This can include information regarding policy content, institutional governance arrangements, global reference points, contextual factors and policy processes (for example, relating to implementation) [42,43]. In practice, this included extracting information from the policy documents that related to these factors, including relevant content, institutional mechanisms, any reference to international agreements, framing (related to context) and the Ministries that led the policy and any other ministries that were named. These considerations also informed the analysis, with respect to our aim to inform policy in the future and in other jurisdictions.

### Data collection

Searches for policy documents relevant to fruits and vegetables at the national and local level were undertaken by systematically searching government websites, and through targeted google searches between July and October 2022. A total of 14 documents were identified for Benin, 18 for Sri Lanka, 30 for Tanzania, and 55 for the Philippines. The search terms were tailored to specific components of the food system and relevant policy sectors. These sectors, identified using food systems frameworks and expert consultation, included Agriculture, Health, Trade & Investment, Commerce, Education, Finance, Water, and Environment, at the national level and in two selected sub-national jurisdictions. Included documents were all publicly available, and these were organized and stored in a shared folder for team use.

A coding framework was developed through an iterative process, grounded in the HLPE food systems framework [21], involving literature review, expert consultation, and pilot testing. The HLPE framework was operationalized by identifying five key domains relevant to fruit and vegetable systems: (1) biodiversity, genetic innovation, and seed systems; (2) sustainable production systems; (3) postharvest and markets; (4) food environments; and (5) consumer behavior. Each domain was further subdivided into specific policy sub-themes with clearly defined indicators to enable systematic coding (see Table 1).

**Table 1:** Coding/extraction framework for specific content relevant to fruit and vegetables.

Policy context	Policy content relevant to fruit and vegetables	Policy (sub)themes
General policy information: policy objectives, activities, ministries named, multisectoral policy provision	Biodiversity, genetic innovation and seed systems	Conservation and use of genetic resources, Crop improvement and plant breeding, Seed systems, seed production, seed policy and seed trade
	Safe and sustainable Production systems	Land use and land management, Water policies & irrigation policies Agricultural subsidies – input subsidies, Extension services for fruits and vegetables specifically, Initiatives for youth participation in production systems
	Post-harvest and inclusive markets	Food Safety, GAP programmes/ policies, Cooperatives, Macro infrastructure (roads etc) and influences on access to markets, Market infrastructure
	Food environments	Retail policy measures relevant to fruits and vegetables, Labeling, School food & nutrition policies & school gardens, Pricing policies relevant to fruits and vegetables,
	Understanding and influencing consumer behavior	Awareness campaigns, F&V in Food Based Dietary Guidelines, Main nutrition priorities, and population groups (high level – obesity/NCDs, undernutrition, micronutrient deficiencies), Initiatives to promote healthy diets/ diet quality re NCD/ obesity prevention, Initiatives to promote healthy diets/ diet quality re undernutrition generally, Initiatives to address micronutrient deficiencies

The framework was operationalized in a structured Excel matrix with explicit coding rules and decision criteria for determining fruit and vegetable relevance: 1. Primary relevance criteria: Policies explicitly mentioning fruits, vegetables, horticulture, or specific fruit/vegetable crops; 2. Secondary relevance criteria: Policies addressing broader agricultural or food system issues that demonstrably impact fruit and vegetable systems (e.g., general irrigation policies, food safety regulations, market infrastructure); and 3. Exclusion criteria: Policies focusing solely on staple crops (cereals, tubers) with no apparent application to horticultural systems

For policies addressing both staple crops and vegetables, content was coded only when specific provisions, activities, or impacts relating to fruits and vegetables could be identified. Where policies mentioned both crop categories, we extracted and coded only the fruit and vegetable-specific content or provisions that clearly applied to horticultural systems.

This matrix utilized both deductive coding based on established food systems theory and inductive coding to capture emergent themes specific to each country context. The excel included a row for each separate policy document and columns for coding.

### Data extraction

The extraction methodology employed a systematic approach with two complementary strategies: First, comprehensive document review involved reading each policy document in full to identify any content meeting the fruit and vegetable relevance criteria outlined above. Second, targeted keyword searches were conducted using terms related to fruits, vegetables, horticulture, and the five food system domains to ensure comprehensive content capture.

Coding was undertaken by reviewing each document line by line, as well as by conducting key word searches to extract data against five specific elements of the food system described above: biodiversity, genetic innovation, and seed systems, sustainable production systems, postharvest and markets, food environments, and consumer behavior. For each policy text segment identified as relevant, coders applied a two-step process: Step 1: Relevance determination – Does this content meet primary or secondary relevance criteria for fruits and vegetables? Step 2: Domain classification – Which of the five food system domains does this content address, and which specific sub-theme indicators apply?

To ensure analytical rigor and minimize subjectivity, we developed detailed coding guidelines (i.e., operational definitions, illustrative examples) with explicit inclusion and exclusion criteria for each category. These guidelines included specific decision trees for handling ambiguous cases, such as policies that mentioned both staple crops and vegetables, or general agricultural policies with unclear applicability to horticultural systems. Each sub-theme contained multiple specific indicators (3–5 per sub-theme) to enable detailed and objective assessment of policy content. Reference points for each of these elements were defined based on the food systems framework and expert consultation within the FRESH research team (see Table 1, ‘policy sub-themes’). These policy sub-themes were designed to reflect key aspects that would need to be addressed within the broader policy content area. We also included codes for cross-cutting policy issues (e.g., equity and inclusion), policy context, resourcing, governance and for policy themes such as framing and beliefs, and implementation (see Table 1).

### Quality assurance and validation

A research lead from each of the countries led coding of policy documents from their jurisdiction. To ensure consistency in applying the fruit and vegetable relevance criteria and coding framework across countries, 20% of policy documents from each country were independently double-coded by another team member. Inter-coder reliability was assessed, and discrepancies were resolved through discussion and refinement of coding guidelines. Text from subset of policies were independently extracted and coded by another member of the team to cross-check the extraction and coding, with regular team meetings to support comprehensiveness and resolve discrepancies. Consultations were conducted with 2–3 key policy stakeholders (under policy divisions of government agencies with focus on fruits and vegetables) in each country to check the completeness of the policy documents and verify interpretation of the policy content. These were conducted iteratively throughout the data collection and analysis. Any additional information to inform the interpretation of content was recorded in a “notes” column in the matrix.

### Analysis

Policy documents were thematically analyzed using the matrix. First, we collated and summarized policy approaches with importance to each food system aspect with relevance to fruits and vegetables, which involved classifying whether policy existed for the specific area; what its major focus areas were; and how it treated fruits and/or vegetables. Second, we summarized sectoral mandates and resourcing to identify policy responsibilities for policies related to fruits and vegetables. Third, we analyzed sectoral framing of fruits and vegetables (how the issue was described, and therefore how policy would suggest tackling it), as well as how gender equality was being addressed by policy. Fourth, a modified SWOT (strengths, weaknesses, opportunities, threats) analysis was conducted to assess policy considerations of fruits and vegetables along the food system in the four study countries, guided by the HLPE food systems framework [21]. The analysis systematically evaluated internal strengths and weaknesses of national policy landscapes, as well as external threats, with reference to specific food system domains: production, distribution, consumption, and food environments. A SWOT analysis aims to evaluate the internal and external factors to aid in crafting strategic plans [44]. Opportunities were then identified with reference to the High Level Panel of Experts [45] framework for food systems and nutrition to assess alignment between national policies and globally recognized pathways for food system transformation. This guided the research team to formulate strategic policy recommendations based on possible combination of internal strengths and weaknesses and external threats and opportunities. Comparison with the framework allowed us to identify key areas where current policies may not fully address all relevant food systems aspects, thereby revealing opportunities to develop more comprehensive fruit and vegetable policy measures that consider multiple dimensions of the food system.

### Research context

The study focused on four case study countries, two in the African region – Benin and Tanzania – and two in the Asian region – the Philippines and Sri Lanka. These four countries are all part of the FRESH (Fruits and Vegetables for Sustainable Healthy Diets) project, a CGIAR initiative led by the International Food Policy Research Institute. All four countries have observed insufficient fruit and vegetable intakes despite significant agricultural production capacity, making them important contexts for identifying systemic barriers and policy opportunities. They also represent diverse agro ecological zones – West Africa, East Africa, Southeast Asia, and South Asia- which enables a rich comparative analysis and cross-contextual learning of fruit and vegetable policy landscapes.

These countries also differ in terms of governance structures (e.g., decentralized vs. centralized), stages of the nutrition transition [46–49], population sizes, urbanization rates, and the extent to which fruits and vegetables are prioritized in national policies. This heterogeneity enhances the analytical depth of the study by allowing exploration of how different institutional, market, and sociopolitical conditions shape food environment policies. However, similar to many LMICs, all four countries have substantial fruit and vegetable production, and face challenges in relation to quality, safety, and postharvest losses affecting both the production and consumption side (see details below). By studying these four countries together, the research contributes actionable insights into how context-specific and shared barriers can be addressed through targeted policy innovations, with relevance to LMICs more broadly.

### Benin

In Benin, agriculture contributed 24% to GDP in 2024 [50], accounted for 83% of export earnings in 2023 [51], and provided employment for 29% of the workforce in 2023 [52]. The country has 31% arable land, totaling approximately 3,545,952 hectares as of 2023 [53]. From 2012 to 2022, production growth rates reached 8.6% for fruits and 80.6% for vegetables, as calculated from FAO [54] data. The Beninese State, in its Strategic Plan for the Development of the Agricultural Sector (PSDSA 2018–2022), has set itself the general objective of contributing to the improvement of the food and nutritional security of the Beninese population and to the increase sustainable contribution of the agricultural sector to the national economy [55].

The agricultural system focuses on cereals (maize, rice), roots and tubers (cassava, yam), and legumes. Major fruits include pineapple, orange, mango, and papaya Tossou, Floquet [56]. Leafy vegetables are widely cultivated across rural and urban areas [57]. Based on 2022 data, the national average daily per capita intake was 87 grams for vegetables and 68 grams for fruits. Vegetable consumption was slightly higher in urban areas (91 g/day) compared to rural areas (84 g/day), while fruit intake was marginally higher in rural areas (70 g/day) than in urban areas (66 g/day) [54].

### Sri Lanka

In Sri Lanka, agriculture contributes 8% of GDP, 11% of export earnings, and employs 26.1% of the active population in 2023 [58–60]. Approximately 1,372,000 ha of land is arable in 2023 [53]. Between 2012 and 2022, production growth rates reached 120.4% for fruits and 43.7% for vegetables [54].

The country cultivates upcountry vegetables (cabbage, carrots, cauliflower) and low-country vegetables (pumpkin, cucumber). Primary fruits include pineapple, mangosteen, papaya, and mango [61]. Daily per capita consumption in 2021 is 124.3g for vegetables and 88.2g for fruits [62], both of which are below the required daily intake. Disaggregated data show that urban populations consume more of both food groups, with fruit intake at 187.78 g/day/person and vegetable intake at 180.55 g/day/person. In comparison, rural areas report lower intakes: 151.51 g/day/person for fruits and 165.89 g/day/person for vegetables [62]. It is worth noting that vegetable consumption is higher than fruit consumption across both urban and rural populations [62]. For the past 30 years, household spending on fruit consumption by Sri Lankan families has increased steadily, but there is a significant disparity between the urban and rural sectors due to higher incomes in the urban sector, which gives them stronger purchasing power [63].

### Tanzania

The sector accounts for 24% of GDP [64], 62% of the export earnings [65], and employs 65% of the workforce in 2023 [52]. As of 2023, the country has 40 million ha of agricultural land, with 15% classified as arable [53]. Most agricultural actors are small-scale subsistence farmers with fewer than four hectares of land and low-tech rain-fed agriculture [66]. From 2012 to 2022, cumulative production growth increased by 26% for fruits and 12% for vegetables [54].

Primary crops include watermelon, oranges, pears, and apples for fruits and beans, cabbages, onions, and tomatoes for vegetables [67]. The FAO data [54] in 2022 indicate a national average daily per capita intake of 111 grams for vegetables and 21 grams for fruits, with urban populations consuming slightly more (vegetables: 119 g/day; fruits: 24 g/day) than their rural counterparts (vegetables: 107 g/day; fruits: 19 g/day).

### The Philippines

In the Philippines, agriculture (including forestry and fishing) contributes to 8.1% of the GDP, 8.7% of total exports, and employs 23.2% of the workforce in 2024 [68–70]. The fruit and vegetable industry has increased in value from 2012 to 2022, with production growth rates of 0.64% for fruits and 7.4% for vegetables [54]. As of 2022, there was an observed increase in pineapple production but declined banana, calamansi, and mango yields [71]. All vegetables and root crops had increased in production except for mongo (−1.5%), cabbage (−0.7%), and ampalaya (−4.3%) [72].

Primary fruits include banana, mango, pineapple, and calamansi while major vegetables comprise ampalaya, cabbage, eggplant, and root crops [71,72]. The Philippines maintains self-sufficiency in vegetables, with Filipinos favoring lowland vegetables over highland varieties [73]. According to the Expanded National Nutrition Survey conducted in 2018–2019, the fruit and vegetable intake of Filipinos declined, and their per capita consumption is less than the recommended 400 grams per day [74]. The mean daily per capita consumption was 58 grams for vegetables and 21 grams for fruits. Vegetable intake was slightly higher among males (61 g/day) than females (55 g/day), and also higher in rural areas (61 g/day) compared to urban areas (54 g/day). Fruit intake was consistently low across all groups, with males consuming 19 g/day, females 23 g/day, and no significant difference between rural and urban populations (both at 21 g/day) [75].

## Results

### Overview

The policy mapping identified relevant policy content within over-arching national plans and strategies as well as within sectoral policies including in the education, agriculture, health, and trade sectors (n = 14 [Benin], 18 [Sri Lanka], 30 [Tanzania], 55 [the Philippines]; see Table 2). A range of policy approaches related to food systems, such as healthy diets, seed systems, production systems, value chains, and food environments, were identified across the countries, which, if implemented effectively, could create a more enabling environment for fruit and vegetable production and consumption.

**Table 2:** Number and types of policy documents analyzed across four low- and middle-income countries.

Type of Document	Benin	Sri Lanka	Tanzania	The Philippines	TOTAL
**National Development Plans**	1		1	1	**3**
**Sectoral policies**					
Health	2	10	5	8	**25**
Agriculture	6	1	11	8	**26**
Commerce & Industry			1	1	**2**
Trade	1	1	2		**4**
Education		1	2	6	**9**
Finance		1	1	1	**3**
Interior and Local Government				2	**2**
Women		1	2	2	**5**
Social inclusion	2		4	1	**7**
**Subnational policies**					
Regional/Provincial		3		3	**6**
Municipal/County/District/City	2		1	22	**25**
**TOTAL**	**14**	**18**	**30**	**55**	**117**

There were significant commonalities in the findings across countries. For instance, multisectoral collaboration, defined in this paper as an organized and intentional effort where diverse sectors and stakeholders join forces in a coordinated process with the aim of achieving common objectives [76], was identified as occurring to some extent in all four countries regarding biodiversity, genetics and seed systems; sustainable production systems; postharvest and markets; food environments; and consumer behavior, though its strength (actual implementation) varied.

In another common finding, we found limited explicit consideration of fruits and vegetables within policies across countries, and some notable gaps in policy (compared to possible policies suggested by the food systems framework) across all policy domains examined at the national level and subnational level. These findings are presented below in Table 3 in a summary of the strengths and weaknesses among the four study countries, S1 Appendix presents a detailed version (including opportunities identified) by country and by elements of the food system.

**Table 3:** Summary of strengths and weaknesses common among Benin, Sri Lanka, Tanzania, and the Philippines.

	Strengths	Weaknesses
**Biodiversity, genetic innovation and seed systems**	Existence of seed policies Emphasis on crop improvement and plant breeding Conservation and protection of indigenous crops and varieties Seed system development and private sector participation Existence of subnational policies and integration with sectoral policies	Lack of sectoral policies on seed systems, seed production, seed policy and trade specifically considering fruits and vegetables Weak multisectoral approach Lack of inclusion in national development plans
**Safe and Sustainable Production Systems**	Sustainable land use and management considerations Water resource management and conservation support Agricultural subsidies and extension services support Youth and women participation considerations Sectoral policies supported by subnational policies	Lack of consideration for safe and sustainable F&V production systems Weak multisectoral approach Weak consideration of marginalized groups other than women and youth Limited focus on fruits and vegetables
**Post-harvest and inclusive markets**	National development plan priorities on food and nutrition security & food safety Sectoral policies on food safety and Good Agricultural Practices Macro and market infrastructure support Subnational policy support Multisectoral approach considered	Lack of consideration for postharvest and inclusive markets for F&V in sectoral and national policies Weak multisectoral approach Lack of support for postharvest technologies and education
**Food environments**	Sectoral policies with explicit considerations for fruits and vegetables Sectoral policies on labeling School food and nutrition policies Food marketing policies Multisectoral approach and subnational policies considered	Lack of consideration for food environments, specifically focusing on fruits and vegetables, in national development plans Lack of sectoral policies on retail measures Weak multisectoral approach
**Consumer behavior**	Awareness campaign/nutrition education specific to fruits and vegetables Focus on vulnerable groups Consideration of nutritional challenges Promotion of healthy diet Existence of policies to meet nutrition objectives	Lack of inclusion in national development plans Weak multisectoral approach Limited reference to food-based dietary guidelines Weak emphasis on fruits and vegetables

### Biodiversity, genetic innovation, and seed systems

Across the four study countries, the policies most relevant to fruits and vegetables included (1) seed system, production, and trade; (2) crop improvement and plant breeding; (3) conservation and protection of indigenous crops and varieties; (4) seed system development and private sector participation; (5) subnational and sectoral policies; and (6) explicit consideration of fruits and vegetables.

All countries had policies in place governing biodiversity, genetic innovation, and seed systems, which are primarily categorized under the agriculture and education sectors, with some policies addressing women’s issues in these contexts. These policies were generally relevant to fruits and vegetables, although they usually did not specify or differentiate between agricultural commodities. There was some variability in priorities and the level of policy used to operationalize these priorities. For instance, Benin had a national seed policy to guide relevant activities for the seed system while in the Philippines, the prioritization of conservation and sustainable use of natural resources was embedded in the National Development Plan. In three countries (Benin, Tanzania, the Philippines), the government had developed a mandate on crop improvement while in Sri Lanka and Tanzania there was explicit policy guidance regarding traditional variety conservation. Moreover, in two countries (Tanzania and the Philippines), there were subnational policies supporting sectoral policies to guide seed systems.

Specific consideration of seed systems relevant to fruits and vegetables was most explicitly identified in Benin, in which there was an explicit policy priority for having continuous availability of fresh fruits through plant breeding, conservation and protection techniques and increasing productivity of factors of production for mango, orange and papaya sector through its National Fruit Tree Development Strategy (SNDAF-2020–2025). In Benin there was also an explicit priority in the national development plan for enhancing the capabilities of its government agencies on production and distribution of vegetable seeds for long-term storage and processing. Moreover, there were specific policy measures in Benin for the adoption of new varieties to increase fruit production through its agriculture sector. Similarly, in Sri Lanka, there was a policy priority for developing climate and pest resistant and high-yielding vegetables. Agriculture sector policies in Sri Lanka and Tanzania had sector-specific explicit considerations for fruits and vegetables in terms of conservation of genetic resources. Sri Lanka had a specific focus on underutilized and neglected fruits and vegetables while Tanzania focused on conservation of indigenous and traditional fruits and vegetables. Tanzania and the Philippines both had subnational policies, at the Provincial or District level that explicitly consider fruits and vegetables, with Tanzania’s subnational policy focusing on training provisions for fruit and vegetable production and processing and the Philippines encouraging the planting of at least 10 fruit trees annually (either household or individual level) to ensure forest protection.

However, the analysis indicated that there was an opportunity for policy to better promote biodiversity, genetic innovation, and seed systems in particular. All four countries lacked sectoral policies specifically focusing on fruits and vegetable seed systems, seed production, seed policy, and seed trade. In Sri Lanka and Tanzania, there was an opportunity for greater inclusion of specific priorities regarding biodiversity, genetic innovation, and seed systems at the whole of government level required to boost national priority and enable the integration of key policy measures across sectors. In the Philippines and Tanzania, there was an opportunity for sectoral policies to include specific policy measures and strategies to address biodiversity and seed systems for fruits and vegetables. Benin’s policies on biodiversity, genetic innovations, and seed systems are addressed as indirect priorities without mentions of specific actions. In Benin’s National Fruit Tree Development Strategy (SNDAF-2020–2025) there are plans to introduce and adapt new species and varieties that could increase fruit production. However, this was without the mention of strategies related to trading of new seed species and varieties.

In relation to governance, there was a gap in multisectoral governance policies on biodiversity, genetic innovation, and seed systems. Tanzania and the Philippines have some multisectoral governance in place in their seed-related policies. However, the description of multisectoral governance in the policies indicated a weak multisectoral approach. It involved mainly the agriculture sector which could be strengthened by also coordinating with trade and finance sectors for investment promotions and relevant market information.

### Safe and sustainable production systems

The documentary analysis indicated that production systems for fruit and vegetables are governed under a wide number of policy documents, including land use and land management policies, water policies and irrigation policies, agricultural subsidies/input subsidies/subsidies and support for export of fruits and vegetables, extension services and youth participation. This was with accountability to a range of sectors, such as agriculture, education, environment, trade, health, and commerce and industry.

All four countries were implementing (1) sectoral policies on sustainable land use and land management, water resource management and conservation; (2) mandates on agricultural subsidies and extension services; (3) initiatives for youth and women participation in production system; (4) sectoral policies supported by subnational policies and some national development plan.

However, variations in policy priorities existed among countries. Benin’s national development plan included priorities on increasing factors of production while the Philippines’ national development plan acknowledged the role of establishing a land use framework to better define land utilization and allocation priorities.

Sector-specific policies for Benin on land management, irrigation, agricultural subsidies, extension services, and initiatives for youth participation in production systems explicitly considered the fruit and vegetable sector through its National Program for the Development of the Vegetable Crops Sub-sector and the National Fruit Tree Development Strategy (SNDAF-2020–2025). Tanzania’s Food and Nutrition Policy (1992) explicitly considered provision of soft loans and agricultural subsidies specifically to fruit and vegetable farmers through its health sector. The Philippines’s RA 8435: Agriculture and Fisheries Modernization Act (AFMA) of 1997 sought to modernize agriculture through investments in irrigation for its high value crops that included fruits and vegetables. Sri Lanka, Tanzania, and the Philippines implicitly supported safe and sustainable production through their agriculture sector policies, with Tanzania and the Philippines having related policies at the local government level. Except Tanzania, all of the three other countries mandated for land ownership and property rights in their agriculture sector policies. Variations in sectoral policies included (1) priority for high value crop production for the Philippines; (2) priority on extension services for farmers engaged in underutilized/neglected food crops with nutritional value (which sector, which country); and (3) the agriculture sector identified as key in higher quality livelihood.

However, within the respective national development plans of all four countries, we did not identify consideration of land management, water & irrigation policies, agricultural subsidies/input subsidies/subsidies & support for export of fruits and vegetables, extension services, and youth participation in production system. Benin had specific sectoral mandates considering fruits and vegetables but these were not reflected in its subnational policies. Sri Lanka, Tanzania, and the Philippines did not have an explicit consideration on fruits and vegetables in their sectoral policies focusing on safe and sustainable production systems. Sectoral policies in Tanzania also lacked mandates on enhancing safe and sustainable production methods in the agriculture sector (National Horticulture Development Strategy & Action Plan 2021–2031, section 2.1.3.7, pg26), had poor agricultural extension and advisory services delivery along the supply chain, and weak support for research–extension–farmer linkages to support demand-driven research and technology adoption [77]. Policies in the Philippines lacked a national land use framework and placed more priority on high value crops that could limit diversification. Moreover, in all four countries, there was limited evidence of a multisectoral approach in agriculture and production-related policies, which involved mainly the agriculture sector with only some involvement of education, health, women, and trade sector.

### Post-harvest and inclusive markets

Relevant policies were identified addressing food safety, GAP programmes, cooperatives or clustering support, and macro- and market infrastructure considerations in national and subnational policies in the four study countries. Sectoral policies spanned across agriculture, health, trade, education, trade, commerce and industry, and women. The key strengths that were similar across the four countries were (1) National Development Plan’s priorities on food and nutrition security & food safety; (2) sectoral policies on food safety and Good Agricultural Practices; (3) sectoral policies on macro and market infrastructure; (4) F&V considerations in sectoral policies; and (5) subnational policy support.

However, there were variations in postharvest and inclusive market considerations across the four countries. Benin’s national development plan indicated a priority for ensuring food and nutritional security and modernizing infrastructure for economic transactions and market access. Whereas the national development plan of the Philippines stated a priority for promoting compliance to international and domestic food safety standards, support for cooperatives, and market infrastructure to prolong postharvest life.

All four countries had sectoral policies focusing on food safety, GAP, support for associations and clusters, and market and macro infrastructure. Sri Lanka’s sectoral policies mentioned support for food safety, good agricultural practices, assistance for cooperatives, and support for macro and market infrastructure through its health and agriculture sector. Moreover, the agricultural sector in Tanzania has been playing a key role in higher quality livelihood.

In the case of three countries (Benin, Tanzania, the Philippines), sectoral policies explicitly address the improvement of post-harvest and market access for fruits and vegetables. However, such explicit mentions are not found in Sri Lanka’s sectoral policies. For instance, in Benin, sectoral policies on fruits and vegetables included a focus on phytosanitary protection of postharvest yields, adherence to production and processing standards, support for association and clusters, credit support for distribution system, enhancement of market access, support for macro and market infrastructure mainly through its agricultural sector which were also translated to subnational policies, without direct consideration for fruits and vegetables. Tanzania explicitly considered safety of fruits and vegetables through regular monitoring by the health sector. Evidence from Tanzania’s food safety control system shows that while the Tanzania Bureau of Standards maintains overarching responsibility for food safety across the production chain, postharvest monitoring faces significant challenges, with inadequate inspection and monitoring along the supply chain linked to foodborne disease outbreaks, and testing conducted episodically by laboratories affiliated with health facilities and regulatory institutions rather than through systematic postharvest surveillance [78]. In the Philippines, agriculture sectoral policies explicitly considering fruits and vegetables included mandates on good agricultural practices for organic and high value crops; and macro and market infrastructure support for high value crops and which were reflected in the subnational policies.

However, in the national development plans of all four countries, there was no explicit consideration on post-harvest and inclusive markets for fruits and vegetables. Sri Lanka’s sectoral policies lacked focus on fruits and vegetables and GAP programs prioritized crops for the export market. Tanzania’s sectoral policies lacked support for cooperative development and production methods did not refer to GAP while the Philippines’ sectoral policies lacked priority for indigenous/local/traditional fruits and vegetables, focus on intensifying better technologies, and provision for education and training on postharvest technologies.

### Food environments

Food environment policies related to retail measures, labeling, school food and nutrition and school gardens, and pricing policies relevant to fruits and vegetables, and included policies led by the agriculture, education, trade, and health sectors.

The strengths observed in all four countries were: (1) explicit considerations of fruits and vegetables in some sectoral policies (2) sectoral policies on retail, labeling, school food and nutrition, and pricing; and (3) a multisectoral approach. For instance, Benin and the Philippines indicate “green” labeling for fruits and vegetables in agriculture and education sector policies, respectively, while Tanzania and the Philippines explicitly consider fruits and vegetables in school gardening for school feeding programs in their education sectoral policy. Retail policy measures in Sri Lanka’s agricultural sector policy mentioned facilitating access to safe, affordable, and quality fruits and vegetables through its health sector while the Philippines has specific agriculture sectoral policies on retail measures for organic produce. Pricing policy measures in Benin under its agricultural sector policy included establishing market price information system specifically for vegetable producers and the Philippines had a subnational policy specifically for highland vegetables at the provincial level. Additionally, the Philippines, in its national development plan, indicated priority for strengthening online marketing and digital transactions and designation of a retail space for organic produce, enhancing food supplementation in schools by providing nutritious food packs (e.g., vegetable-based noodles), and intensifying food price monitoring. Furthermore, Sri Lanka had specific policy mandates on food labeling, school food nutrition, pricing standards for staples, price control specific for fruits and vegetables, and regulation of food promotions in schools

However, in all four countries, national development plans had not explicitly considered the aspect of food environments in relation to fruits and vegetables, except for the Philippines mentioning enhancing of food supplementation in schools through provision of vegetable-based products through its education sectoral policy. Benin’s labeling policy is mainly for the fruit tree sector and lacks provision of inclusion of mandatory information. In the Philippines, green labeling only applies in school canteens. In 3 countries (Sri Lanka, Tanzania, the Philippines), there is a lack of mandate for food marketing and in the Philippines, food marketing regulation is only at the subnational level and only focusing on organic and highland vegetables. Moreover, Tanzania’s school feeding programs are mainly for children with HIV. There is also a weak multisectoral approach, involving only some sectors in food environment-related policies. For example, food environment-related policies in the Philippines and Tanzania primarily span education, health, and agriculture. In contrast, Sri Lanka’s policies involve the agriculture and health sectors, and Benin’s policies encompass agriculture, trade, and education sectors.

### Consumer behavior

Sectoral policies relevant to fruit and vegetable consumption were identified in agriculture, health, trade, women, and education. The policies related to awareness campaigns, food-based dietary guidelines, nutrition priorities and population group, promoting a healthy diet, and micronutrient deficiencies. Sectoral policies analyzed encompasses agriculture, health, trade, women, and education. Across the four countries, key strengths in policies analyzed include: (1) considerations on raising awareness and provision for nutrition education with explicit consideration on fruits and vegetables; (2) focus on vulnerable groups (children, women, and adolescents) to address nutritional needs; (3) emphasis on targeted nutrition challenges; and (4) existence of sectoral policies to guide the countries’ nutrition-related efforts.

In terms of fruit and vegetable considerations in relation to consumer behavior, variations were found in the way fruits and vegetables were being promoted to consumers. For instance, in the Philippines, there was an explicit consideration in its National Development Plan of a multisectoral approach to promote healthy diet through nutrition-sensitive programs such as school vegetable gardens. In Benin, there were awareness campaigns for fruits and vegetables across the agriculture sector and subnational level. In Sri Lanka, Tanzania, and the Philippines, awareness campaigns included promotion of healthy food intake that included fruits and vegetables through school curricula and the media. Three countries national dietary guidelines are in place promoting fruits and vegetables as a core group. However, Sri Lanka was the only country with active promotions of food-based dietary guidelines in its National Nutrition Policy, with recommendations to increase vegetable consumption and promotion of safe and quality fruit production through the agriculture sector. Initiatives to promote healthy diet in Sri Lanka, Tanzania, and the Philippines emphasized fruit and vegetable consumption, with Sri Lanka’s health and education sectoral policies having specific mentions of green vegetables and leafy greens.

However, key common weaknesses in policies included: (1) a lack of inclusion in national development plan on key strategies for consumer behavior and preferences relevant to fruits and vegetables; (2) limited reference to food-based dietary guidelines; (3) a limited multisectoral approach on consumer aspects; and (4) a weak emphasis on fruits and vegetables in most policies analyzed. In all four countries, it is evident in the policy documents analyzed that some initiatives to promote healthy diets/diet quality in relation to undernutrition, NCDs, and micronutrient deficiencies existed, with awareness campaigns to promote healthy lifestyle and with nutrition priorities spanning various age groups. However, we found that among the three countries that had food-based dietary guidelines in national nutrition policies, Benin, Sri Lanka and the Philippines, these were not consistently referenced in other sectoral policy documents. Furthermore, the countries examined in the analysis did not comprehensively address all three areas, namely noncommunicable diseases (NCDs), undernutrition, and micronutrient deficiencies. We also noted a weak multisectoral approach encompassing mainly agriculture, health, trade sectors, and some women and education sectors as well as a weak emphasis on fruits and vegetables in all sectoral policies.

## Discussion

Our analysis of the policy landscape in the four LIMCs, guided by the Food Systems Framework and theories of policy learning, indicated that there were specific food system policy opportunities in Agriculture, Health, Environment, Trade, Education, and Commerce and Industry policy sectors that could improve fruit and vegetable production, availability and consumption. However, while there is strong rhetoric for improving nutrition and health across governments, including through increasing fruit and vegetable consumption, there was a limited explicit consideration for fruits and vegetables in most major policies governing food systems. The Food Systems Framework emphasizes interconnections between production, distribution, and consumption, yet our findings show these links are poorly reflected in existing policies regarding fruits and vegetables. The governments of the four countries examined were implementing a substantial number of strategies that have the potential to improve fruit and vegetable availability, accessibility and consumption, but only a few of these are explicitly targeting fruits and vegetables. While such specificity in policy may not be essential – for example, due to common agricultural inputs and market-related infrastructure with other key foods – fruits and vegetables do have some very specific attributes (in terms of production, trade, and consumption) that mean they need to be considered explicitly in many cases, and the pervasive challenge of affordability and accessibility of fruits and vegetables for consumers indicate that more specific policy measures are likely to be needed to change the status quo [6,79]. Such measures can also contribute to addressing related environmental priorities regarding climate change and biodiversity conservation [80].

In reflecting on the findings of this analysis in light of existing literature, five key strategies are discussed below that these and other LMIC countries facing similar challenges could pursue to transform the food system to include fruits and vegetables and promote better health outcomes. Additionally, it is important to take into account the interconnections within the food system when considering these strategies.

### Policy prioritization for fruits and vegetables

Though we noted strong priority for promoting nutrition and health in policies, particularly in national development plans, we found only a few occasions where food systems policy placed explicit emphasis on addressing inadequate fruit and vegetable consumption as a way to promote nutrition and health. The low fruit and vegetable intake in LMICs can be addressed in part through policies and programs [81]. To do this, countries need to reorient public resources and policies which are currently focused on economic productivity alone (and therefore often focus either on staple grain, cash crops, or export crops) [82,83] to also include attention to making a healthy diet available within each country, and not neglect the value in diets of traditional fruits and vegetables and those which are often not produced commercially [84].

Several countries in Latin America have demonstrated how fruit and vegetable policies can be effectively integrated into broader food systems strategies. For instance, Brazil’s Programa Nacional de Alimentação Escolar (PNAE) mandates the use of locally sourced fruits and vegetables in school feeding programs, which has not only improved dietary diversity but also stimulated smallholder production [85]. In Chile, while the Food Labeling and Advertising Law primarily targeted highly processed foods, one study found spillover effects on fruit purchasing—an increase in the likelihood of fruit purchases and with effects more pronounced among college-educated and low-income households [86]. These cases show that well-designed policies can lead to measurable improvements in diet quality and should inform cross-regional policy learning.

To address the lack of fruit and vegetable considerations in food system policies, the desired dietary outcomes should be explicitly considered in policy development, alongside food security, income, and other food system outcomes [87,88]. National policies should reflect a recognition of the role of fruits and vegetables in addressing poor diet and malnutrition issues, which have been established by previous studies [14–16]. However, this recommendation should consider national contexts. In the case of Sri Lanka with a centralized governance structure [89], national-level mandate can drive prioritization of fruits and vegetables but should consider mechanisms for subnational adaptation. Conversely, Benin, the Philippines, and Tanzania have decentralized governance models [90–92] that can facilitate localized innovation but require empowering subnational government with resources and specific guidelines to support context-specific interventions. Economic disparities and cultural dietary preferences, such as stronger reliance on traditional leafy vegetables in Benin [93] should inform targeted investment, education campaigns, and inclusive market development strategies.

There is also an opportunity for national development plans to provide a framework for addressing important aspects of food systems relevant to fruits and vegetables, to guide sectoral policies. These include: (1) biodiversity and seed system with particular consideration for crop productivity, sustainability, and resilience; (2) safe and sustainable production systems; (3) postharvest and inclusive markets; and (4) healthy food environments with specific focus on fruits and vegetables.

The study findings indicate an opportunity for sectoral policies to better support fruits and vegetables through increasing investment in crop diversification. Because there has been a strong focus on staple crops in most policies reviewed, it will be beneficial to explore opportunities for strengthening crop diversification and biodiversity on the upstream of the fruit and vegetable food system. Improving traditional varieties and knowledge dissemination in sectoral policies might offer valuable paths to enhance the quality and acceptability of fruits and vegetables in food supply chains.

Policies should extend beyond the crop and production stages of food system and across into the food system. The midstream of the fruit and vegetable food system should also be enhanced by strengthening policies on processing and preserving innovations. While policy actions to strengthen post-harvest management and improve access to markets exist in the policies, they seldom targeted fruits and vegetables, food safety and Good Agricultural Practices (GAP), enhancing human capacities through GAP and food safety trainings, or better support for appropriate postharvest infrastructure. Moreover, on the downstream side, sectoral policies should reflect prioritization of availability and promotion of fruits and vegetables through labeling, marketing strategies, and price control mechanisms. We noted that in most countries, schools had been identified as setting to interact at all stages of production, processing and consumption. However outside of schools, there appeared an overemphasis on consumer behaviors to promote fruit and vegetable consumption, rather than structural changes to food environments. Promotion of healthier food consumption through nutrition education and communication strategies should be strengthened and should refer to food based dietary guidelines, which could influence dietary patterns [5], highlighting the critical role of fruits and vegetables in the diet.

### Multisectoral collaboration

Drawing on a food systems framework to analyse policies highlighted the importance of multiple sectors in supply chains and food environments relevant to fruits and vegetables and identified a need to strengthen multisectoral collaboration, based on policy gaps that revealed overlapping sectoral responsibilities. The analysis showed that certain mandates would benefit from joint ownership across sectors, reflecting the multistakeholder nature of food systems where policies in one sector inherently impact and require coordination with others.

Among the four study countries, only Tanzania had explicitly considered a multisectoral approach in its national nutrition plan [94]. Tanzania’s implementation experience demonstrates practical intersectoral coordination through government officers’ engagement in community and intersectoral collaborations, which has provided actionable guidance for translating national multisectoral nutrition (MSN) policy into practice while utilizing implementation staff capacity [95]

The research identified a range of instances in which more than one policy sector was explicitly mandated to work together. For instance, the National Strategic Plan on Child Health of Sri Lanka states a mandate that the Family Health Bureau will work with the Ministry of Education to improve the health of school children [96]. Benin’s national program for the development of the vegetable crops sub-sector [97] involves agriculture, trade, and transportation sector. However, although these sectors are indeed relevant and appropriate, there is an opportunity to use a food systems lens to consider potential involvement from other related sectors, such as environmental protection and public health, to create a comprehensive and holistic approach. Evidence from Benin demonstrates strong government commitment to multisectoral coordination leading to the establishment of the Food and Nutrition Council (FNC) under the President’s auspices that brings together agriculture, health, social protection, finance, planning, decentralization, industry sectors, national associations, academia and civil society to develop and coordinate multisectoral nutritional policies and programs [98]. Notably, the creation of a National Agency for Food and Nutrition in Benin through the decree n°2023−425 of July 26, 2023 whose mission is “to ensure food and nutritional security by promoting healthy eating, improving the nutritional status of the population and preventing diet-related diseases” presents a promising institutional entry point for advancing this multisectoral agenda.

Improved coordination among multiple sectors is essential to promote production, distribution, and food environment factors specific fruits and vegetables. Addressing low fruit and vegetable production-consumption necessitates integrated solutions spanning across various sectors, though the extent of sector involvement should be tailored to the specific context and challenges. A multisectoral strategy integrates a comprehensive set of approaches in a cohesive manner [99]. For instance, in Sri Lanka’s hierarchical administrative structure, coordination could be enhanced through mechanisms such as cabinet committees, the appointment of cross-sectoral junior ministers or ministers without portfolio, and priority-driven program management systems that compel inter-ministerial collaboration [100]. Tanzania’s established multisectoral nutrition committees provide a foundation for fruit and vegetable promotion yet effective implementation necessitates that local actors move beyond sectoral silos [95], a challenge similarly observed in other decentralized governance systems such as those in the Philippines [101] and Sri Lanka [102].

Moreover, to ensure sustainability, food system policy choices need to address multiple outcomes (e.g., nutrition, health, environment) [103]. In order to effectively align actions and drive transformative change, engaging actors at multiple scales is necessary [104] as health issues themselves may be spillovers from actions of other sectors [105]. Although multisectorality is difficult in practice due to sectoral mandates, structures, and power relations [106], national development plans can play an important role in clearly defining the involvement of various sectors (as appropriate) that could facilitate comprehensive planning and budgeting at the sectoral level [107].

### Policy integration across levels

Policy integration refers to an approach to managing issues in policy-making that are cross-cutting with respect to established policy sectors, in which an issue is comprehensively addressed through multiple policy instruments across multiple sectors [108]. Our findings suggest that beyond multisectoral considerations, further policy integration across levels might be warranted. We noted that in all study countries some subnational policies exist. However, sectoral policy design and implementation could further be strengthened through integration of priority for fruits and vegetables across national government and decentralized decision-making. This would also enable local adaptation and accountability [109]. For instance, in this study, we identified opportunities at the national government level in the Philippines, suggesting the potential for additional opportunities at the local government level. Reflecting on the legislative framework, the Local Government Code of 1991 in the Philippines declares that subnational governments are partners in the attainment of the national goals [90], thereby acknowledging the role of local governments in contributing to these efforts. Therefore, the local government provides complementary functions to the national government in formulating region-specific strategies, bridging policy gaps or limitations, and establishing region-specific administrative structures [110], ensuring that policies are customized to local situations [111].

The potential for collaborative enhancement in the agriculture sector is exemplified by initiatives in Sri Lanka, where the national mandate emphasizes the establishment of a market-oriented agricultural system. This is complemented by a subnational directive on developing infrastructure facilities to enhance production and productivity of food crops in provinces. By working in synergy, the national and subnational level can contribute to the overall improvement of the agricultural sector. The extent of integration, however, can be constrained by the degree of autonomy of the subnational government. For instance, in Sri Lanka, central institutions dominate [89] which could potentially affect collaborative efforts. On the other hand, countries like Benin, Tanzania, and the Philippines operate in a decentralized manner where subnational governments have authority for executive and legislative responsibilities [90–92]. Yet, it is also important to recognize that lower degree of integration may also be a practical approach to manage cross-cutting issues [112].

Moreover, to ensure effectiveness, policy integration across levels must also be responsive to existing economic and cultural contexts. In Sri Lanka, for example, integration strategies should strengthen the existing market-oriented agricultural infrastructure while recognizing traditional home gardens as culturally significant entry points for promoting fruits and vegetables [113]. In Tanzania, integration must address district-level resource constraints [114] through phased implementation in horticultural regions while building on cultural values around indigenous vegetables [115]. Benin’s integration strategy could be bridging formal and informal agricultural markets while respecting traditional leafy vegetables’ cultural significance [116]. In the Philippines, integration across levels should account for economic constraints faced by local governments, as well as systemic challenges such as limited administrative capacity, fragmented service delivery, and the influence of local political dynamics on implementation efficiency [117].

### Sustainability and resilience

Most of the policies reviewed could further be enhanced to explicitly consider sustainability and resilience of fruit and vegetable food systems to address system-wide vulnerabilities from production to consumption. The Food Systems Framework highlights how food system activities must sustain both environmental and human and institutional capacities while building resilience to withstand disruptions. Our findings show that current policies inadequately address these outcomes specifically for fruits and vegetables, despite their unique vulnerabilities in production, distribution, and consumption.

At the upstream level, this could be achieved by strengthening seed quality assurance systems that ensure the availability of climate-resilient, pest-resistance, and high-yield fruit and vegetable varies. This can be operationalized through three key steps: (1) establishing or upgrading national seed certification agencies to regulate seed standards; (2) supporting public–private partnerships to develop and disseminate quality seed for underutilized and indigenous vegetables; and (3) training local seed producers and agricultural officers on varietal purity, germination testing, and disease resistance screening [118–120]. Seed quality assurance ensures that seeds produced and distributed create better yields and reduce losses and can adapt to changing climate conditions [121]. Access to high quality seeds is a major challenge in LMICs [122,123]. Second, enhancing human capacities of local farmers and experts on biodiversity and biotechnology can be achieved through targeted training programs in biodiversity conservation, participatory varietal selection, and biotechnology applications in vegetable or fruit breeding. A strong human capacity on biotechnology can aid promotion of diverse and locally adapted varieties resilient to local condition. Third, encouraging fruit and vegetable crop diversification, including both improved and traditional or locally-adapted seed, can also reduce risks of crop failure due to extreme weather events [124].

At the midstream level, strengthening the research–extension–farmer linkages can facilitate demand-driven research and adoption of technology through a bidirectional exchange of information [125]. This may also improve the chain’s resilience, as farmers need to utilize timely coping strategies (e.g., land management practices, agricultural techniques, use of specific crop types) against climate change impacts [126]. There is also a need for enhancing infrastructure support and human capacities for quality assurance at post-production/postharvest sites specifically for fruits and vegetables, to optimize the food system through reduced postharvest losses, improved quality, consistent supply, and enhanced reputation for producers and the whole food system.

At the downstream end, we see the need for strengthening price control mechanisms to include fruits and vegetables as basic commodity to ensure consistent access to fruits and vegetables even during disruptions and reduce food waste through increased consumer demand [127] alongside reinforcing communication strategies and nutrition education to encourage individuals and households to prioritize fruits and vegetables, leading to increased demand even during disruptions.

Moreover, approaches to sustainability and resilience should tailor to national contexts. For instance, in Sri Lanka, the dual challenges of monsoon flooding and drought necessitate climate-adaptive cultivation systems that integrate traditional water management practices with modern technologies [128], while also addressing economic constraints following the recent financial crisis [129]. In Tanzania, improving land tenure security [130] can encourage long-term investments in sustainable horticultural practices. In Benin, policy strategies can build upon traditional polyculture farming systems [131] that inherently demonstrate resilience principles, while also addressing economic barriers to agricultural production. In the Philippines, where vulnerability to typhoons and other climate-related disasters is high, commons-based approaches that strengthen agro ecological resilience among smallholder farmers is recommended [132].

### Inclusion and equity

Our study further suggests opportunities for policies relevant to fruits and vegetables to better consider the needs and challenges of marginalized communities, small-scale farmers, and women, with an explicit aim for equity and inclusivity [133], thus ensuring policy benefits reach marginalized producers and consumers. Current policies inadequately address equity issues such as land tenure security, market access for small-scale producers, and gender-specific barriers. These gaps affect both production and consumption patterns, particularly among vulnerable groups, demonstrating the need for more targeted policy interventions to achieve equitable food system outcomes.

At the upstream level, quality seed production and distribution for small-scale fruit and vegetable farmers jointly with agricultural extension services at the local government level could be enhanced; alongside ensuring that traditional seed systems are protected, particularly for indigenous vegetables [134]. A functional and inclusive agricultural system is necessary to address information gaps and enable the spread of agricultural innovations and the support of different population groups [135]. Our review also suggests strengthening access to land, through land tenure, which plays a critical role in empowering and improving diets and nutrition of smallholders, rural communities, and indigenous peoples [103]. Land tenure security influences allocation of land for various purposes, availability of resources, and type of investments for land conservation and productivity [136]. In Tanzania, women’s land property rights lack adequate legal protection due to persistence of customary laws [137]. Evidence in Sri Lanka suggests that land tenure differences influence land investment behavior of farmers [138] and production planning, which could then lower investments in food production [103].

At the midstream level, we suggest an inclusion of support and regulation mechanism for fruit and vegetable traders, as they enhance smallholder commercialization [139]. At the downstream level, this points to an opportunity for a policy provision for establishing fruit and vegetable markets with specific pricing mechanisms to increase fruit and vegetable intake in low-income areas [140,141]. Enhancing market access for fruits and vegetables in low-income areas requires a tiered policy approach that addresses both the supply and affordability constraints faced by marginalized consumers. This can be done by first designating and developing community-based fruit and vegetable markets in underserved urban and peri-urban neighborhoods [142]. Second, targeted pricing policies, such as subsidized vendor stall fees, price ceilings on essential vegetables during shocks, or consumer vouchers for low-income households, can improve affordability and reduce seasonal price volatility. Third, inclusive procurement policies can link smallholder vegetable producers—especially women and informal traders—to public institutions such as schools and hospitals [143], as these can provide steady demand, reduce marketing risks, and promote equity across the supply chain.

Moreover, inclusion and equity strategies must be tailored to each country’s distinct sociopolitical context. For instance, addressing the economic marginalization of Tamil communities [144] requires targeted fruit and vegetable programs that overcome historical exclusion while providing culturally appropriate extension services. In Tanzania and Benin, equity strategies must address gender-based barriers in land tenure [145,146], which limits women’s participation in fruit and vegetable production. In the Philippines, policies should prioritize safeguarding access to traditional lands for indigenous communities to support the cultivation of native fruit and vegetable varieties and prevent further socio-economic dislocation resulting from plantation expansion and inequitable land deals [147].

This study makes several contributions to the literature on food systems approaches to nutrition policy. As highlighted in previous study, effectively improving public health necessitates adopting a comprehensive food systems policy approach [148]. Building on this premise, our study contributes by providing a cross-country comparative analysis that identifies specific gaps in policies that systematically disadvantage fruit and vegetable promotion across diverse LMIC contexts. This study advances existing literature by applying a comparative, fruit-and-vegetable-specific lens across multiple food system domains, identifying concrete policy entry points and sector-specific gaps, and demonstrating how broad food system recommendations can be operationalized in diverse governance and agricultural contexts. By applying a structured analytical framework to evaluate policy alignment across agricultural, health, trade, and environmental sectors, we move beyond theoretical food systems approaches to provide actionable, context-specific entry points for policymakers. Our findings reveal that the primary obstacles to effective fruit and vegetable policy are not merely the absence of relevant policies, but rather insufficient cross-sectoral coordination, limited subnational adaptation, and inadequate crisis resilience mechanisms—insights that significantly extend current understanding of fruit and vegetable policy implementation in resource-constrained settings.

## Conclusion

Our comparative policy landscape analysis, grounded in a food systems framework and informed by theories of policy learning, identified limited explicit consideration for fruits and vegetables in most of the policies reviewed governing food systems in Benin, Sri Lanka, Tanzania, and the Philippines despite the existing strategies with the potential to improve fruits and vegetable availability, accessibility, and consumption.

We recommend that policy sectors explicitly consider fruits and vegetables across the food system from production to consumption when drafting policy, with national development plans explicitly considering the significant role of fruits and vegetables in addressing malnutrition and food insecurity issues, and prioritizing approaches to promote their consumption. There is also a need for oversight across sectors, to ensure that policies strategically complement each other to enhance production, distribution, and consumption of fruits and vegetables. Moreover, policy integration (better communication) between the national and subnational levels can enhance implementation of policies with due consideration of the local context. Sustainability and resilience of fruit and vegetable supply chains should be strengthened through integrated policy measures—such as improving seed quality assurance and local biotechnology capacity, expanding postharvest infrastructure and cold chains, and classifying key fruits and vegetables as essential commodities during crises. These actions would help ensure consistent availability and affordability of fruits and vegetables even in times of disruption such as climate shocks or public health emergencies like the COVID-19 pandemic. To promote equity and inclusion in fruit and vegetable policies, governments should strengthen land tenure security for smallholders—especially women—support traditional seed systems for indigenous producers, and provide targeted investment in extension services and local markets. Moreover, incorporating pricing mechanisms for fruits and vegetables in low-income areas and formalizing support for informal traders can help ensure that marginalized producers and consumers both benefit from a more inclusive and nutritious food system. Future research should focus on how these recommendations could be achieved, given the varied political and economic contexts among LMICs. Given the diversity of the countries studied, these results are likely to be highly relevant to other countries seeking to improve fruit and vegetable supply and consumption.

This study has several limitations. First, its primarily qualitative approach to policy analysis limits our ability to provide statistical comparisons or definitive rankings of policy effectiveness across countries. Moreover, while the policy document review provided valuable insights into the formal policy landscape, it may not fully reflect implementation realities or informal practices that influence food systems on the ground. Policy documents also vary in scope, detail, and accessibility across countries, which may have led to uneven depth of analysis.

In addition, the use of a modified SWOT analysis, though useful for synthesizing cross-country policy strengths, weaknesses, and opportunities, is inherently qualitative and subject to interpretation. Although the analysis was structured using the HLPE food systems framework and supported by policy documents, it did not rely on systematic quantitative indicators across all countries. As such, findings should be understood as exploratory and indicative rather than conclusive. Future research should complement qualitative insights with more robust quantitative policy performance metrics and examine how the recommended policy shifts could be operationalized in diverse LMIC contexts.

Moreover, the study did not investigate in depth the political, institutional, or historical factors influencing the formation of policies or the barriers to their implementation. Future research could build on this study by incorporating qualitative case studies, stakeholder interviews, and political economy analysis to more comprehensively examine the processes that shape fruit and vegetable prioritization in LMIC food systems. Moreover, employing mixed methods or econometric approaches to quantitatively assess the impact of specific policies on fruit and vegetable production and consumption outcomes would allow for deeper exploration of causal relationships and policy effectiveness across different contexts and could further strengthen the evidence base. Finally, while this study provides system-level recommendations, we recognize the need for future research that incorporates political economy analysis and cross-country case studies to more concretely identify implementation pathways and context-specific response measures tailored to different national governance structures

Nonetheless, the diversity of countries included suggests that these findings may be relevant to a wide range of settings seeking to strengthen fruit and vegetable policies through a food systems approach.

## Supporting information

S1 Appendix Suggested opportunities to improve policy design in four study countries (Benin, Sri Lanka, Tanzania, and the Philippines). (DOCX)
