# Supplementary material for: Policy landscape analysis for fruits and vegetables in four low- and middle-income countries through a food systems approach
Source: PLoS One. 2025 Sep 19;20(9):e0331287. doi: 10.1371/journal.pone.0331287 (PMC12448988; doi:10.1371/journal.pone.0331287)
Supplement: S1 Appendix — (DOCX) [file pone.0331287.s001.docx]

**Appendix A. Suggested opportunities to improve policy design in four study countries (Benin, Sri Lanka, Tanzania, and the Philippines).**

|  | **Policy prioritization for fruits and vegetables** | **Multisectoral collaboration** | **Policy integration** | **Sustainability and resilience** | **Inclusion and equity** |
| --- | --- | --- | --- | --- | --- |
| **Biodiversity, genetic innovation and seed systems** | - Inclusion in the national development plan key strategies for biodiversity, genetic innovation, and seed system focusing on fruits and vegetables with particular consideration for crop productivity, sustainability, resilience, and adaptation - Strengthen infrastructure support for fruit and vegetable (F&V) biodiversity and seed systems - Consider improvement of traditional varieties, knowledge dissemination of improved varieties, or indigenous fruits and vegetables - Creation of conducive environment for seed production and trade through promotion of plant breeding activities and Intellectual property rights | Enhance multisectoral collaboration | - Strengthen sectoral and subnational policies to include priority on fruits and vegetables - Build on existing initiatives at subnational level to regulate seeds and support seed systems and biodiversity, by scaling up support for community-based seed production systems for seed requirements at the local level | - Strengthen seed quality assurance regulation - Enhance human resource capacities through trainings of local experts on biodiversity and biotechnology | - Enhancement of seed production and distribution chain for the fruit and vegetable industry, with specific considerations for small scale farmers and women - Enhance farmers’ knowledge on improved varieties |
| **Safe and Sustainable Production Systems** | - Inclusion in the national development plan key strategies for safe and sustainable fruit and vegetable production systems to guide sectoral policies - Promote crop diversification to include priority on underutilized or neglected or indigenous fruits and vegetables - Promote productive farming systems such as crop prioritization (prioritization over diversification?), crop zoning, precision agriculture - Promote agricultural efficiency through better technology and investment in agriculture human capital development along the supply chain | - Enhance multisectoral approach and subnational policy support | - Strengthen sectoral and subnational policies to include priority on fruits and vegetables | - Address key issues on fruit and vegetable production systems in relation to climate change - Strengthen research–extension–farmer linkages to support demand-driven research and technology adoption | - Strengthen land rights |
| **Post-harvest and inclusive markets** | - Inclusion in the national development plan key strategies for postharvest and inclusive markets for fruit and vegetables to guide sectoral policies - Enhance support for appropriate infrastructure - Enhance human capacities on GAP and food safety through trainings - Strengthen policies on food safety and GAP to cover fruits and vegetables for the local and international market | - Enhance multisectoral approach and subnational policy support | - Strengthen sectoral and subnational policies to include priority on fruits and vegetables | - Enhance infrastructure support and human capacities for quality assurance at post-production/postharvest sites specifically for fruits and vegetables – to optimize food system & minimize postharvest losses |  |
| **Food environments** | - Inclusion in the national development plan key strategies on healthy food environment with specific focus on fruits and vegetables to guide sectoral policies - Inclusion of retail policies (e.g. incentives) to encourage retailers to offer fruits and vegetables, and establish fruit and vegetable markets in low-income areas - Inclusion of policies on incentivizing food retailers and restaurants to offer and promote consumption of fruits and vegetables - Inclusion of labeling mandates to promote fruits and vegetables through green labeling - Inclusion of food marketing regulations relevant to fruits and vegetables, such as placement strategies and price promotions - Strengthen price control mechanisms to include fruits and vegetables as basic commodity | - Enhance multisectoral approach | - Strengthen sectoral and subnational policies to include priority on fruits and vegetables | - Strengthen price control mechanisms to include fruits and vegetables as basic commodity and ensure equitable access to fruits and vegetables | - Inclusion of retail policies (e.g. incentives) to encourage retailers to offer fruits and vegetables, and establish fruit and vegetable markets in low-income areas |
| **Understanding and influencing consumer behavior** | - Inclusion in the national development plan key strategies to influence consumer behavior through promotions of fruit and vegetable intake - Strengthen communication strategies and nutrition education to promote consumption of fruits and vegetables - Use food-based dietary guidelines as reference to promote healthier food consumption, highlighting the role of fruits and vegetables in the diet - Strengthen initiatives or mandates to promote healthy diets/diet quality to address NCD/obesity prevention, undernutrition, and micronutrient deficiencies through fruit and vegetable consumption | - Utilize a multisectoral approach to nutrition-related components of fruits and vegetables and augmented by subnational policies | - Strengthen cross-sector operationalization of the right to adequate nutrition, including fruits and vegetables | Strengthen communication strategies and nutrition education to promote consumption of fruits and vegetables | - Strengthen communication strategies and nutrition education to promote consumption of fruits and vegetables - Strengthen cross-sector operationalization of the right to adequate nutrition, including fruits and vegetables |
